# Design of organic structure directing agents to control the synthesis of zeolites for carbon capture and storage†

**DOI:** 10.1039/c9ra09675b

**Published:** 2019-12-17

**Authors:** Frits Daeyaert, Michael W. Deem

**Affiliations:** Department of Bioengineering, Rice University Houston TX 77005 USA mwdeem@rice.edu; FD Computing Stijn Streuvelsstraat 64, 2340 Beerse Belgium; Department of Physics & Astronomy, Rice University Houston TX 77005 USA

## Abstract

We have *de novo* designed organic structure directing agents (OSDAs) for zeolites that have been predicted to be effective materials for carbon capture and storage. The zeolites were selected for their reduced parasitic energy when used as CO_2_ adsorbants in a pressure–temperature swing process in coal- or natural gas-fired power plants. Synthetically accessible OSDAs were designed for five known and two theoretical frameworks.

## Abstract 2

Zeolites more efficient at carbon capture than scrubbing with aqueous monoethanolamine, and the best OSDAs designed for the synthesis of each.

## Graphical abstract

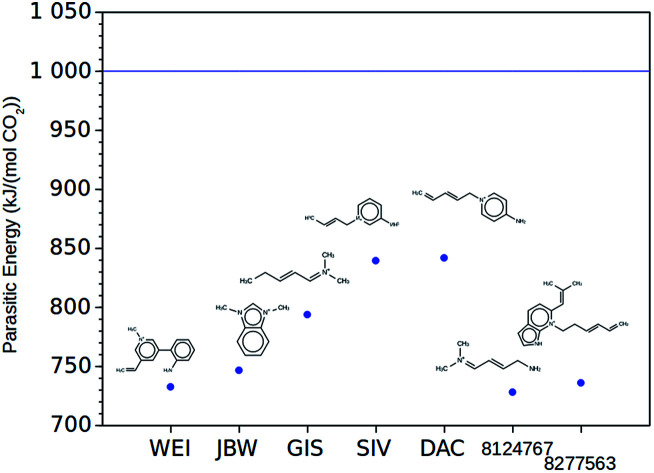

## Introduction

Carbon capture and storage (CSS) in fossil fuel power plants^1^ is considered the most economically feasible strategy for mitigation of global warming by carbon dioxide emission.^1^ The most mature CSS technology currently is scrubbing the CO_2_ produced in the fuel combustion process with aqueous monoethanolamine.^2^ This process is highly energy consuming.^3^ Therefore alternative technologies, including adsorption of CO_2_ in nanoporous materials such as zeolites and metal–organic frameworks, are the subject of numerous studies.^4^

Zeolites are meta-stable forms of alpha-quartz and are built up from tetrahedral TO_4_ building blocks, where T is generally Si or Al.^5^ The specific arrangement of the building blocks leads to zeolite frameworks characterized by nanopores of various sizes and shapes. To date, 248 different zeolite frameworks have been identified.^6^

**Fig. 1:**
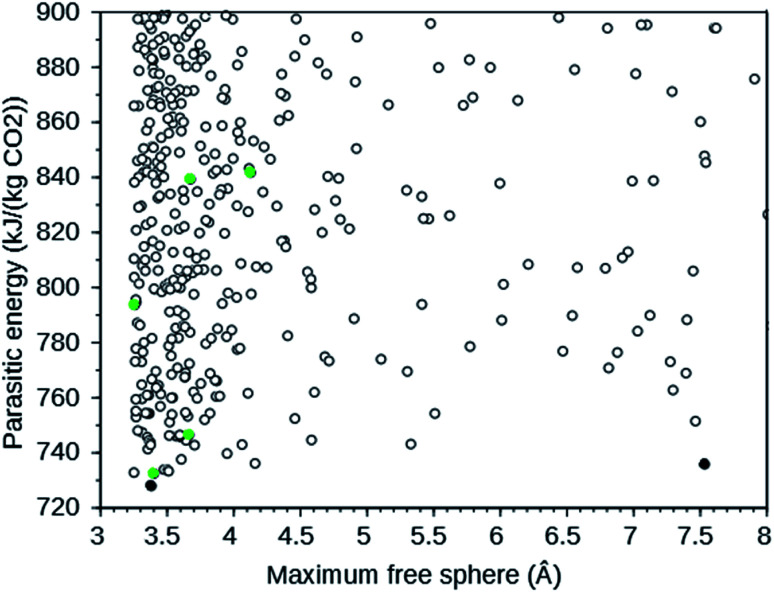
Parasitic energy *versus* maximum free sphere diameter of IZA (green) and theoretical (black) zeolite frameworks screened by ref. 7. The plot is cut off at a parasitic energy of 900 kJ (kg CO_2_)^−1^ and a maximum free sphere of 8 Å. The filled circles are the target frameworks we selected. The data were downloaded from ref. 9.

A large-scale computational screening effort to identify existing and theoretical zeolite frameworks has been undertaken.^7^ Zeolite frameworks were identified that absorb CO_2_ with minimal parasitic load on a power plant. Both the energy needed in a temperature–pressure swing capture and in compression of the separated CO_2_ were considered. The parasitic energy of the CSS process is the sum of the energy required to heat the material for the temperature swing, the energy required to balance the heat of adsorption, and the energy required to pressurize the CO_2_ to 150 bar for transport and storage. Since flu gases are typically 75% N_2_, 12–15% CO_2_, 10% H_2_O, and 3% O_2_ at 40 °C and 1 atm,^7^ the separation of N_2_ and CO_2_ is the dominant one. Existing zeolites from the IZA database^6^ and a selection of theoretical frameworks with large enough pore diameters from a database with over 331 000 entries^8^ were screened. The following IZA frameworks were predicted to have the lowest parasitic energy: WEI, JBW, GIS, SIV, and DAC. In addition, multiple theoretical frameworks were predicted to have a lower parasitic energy than any of the IZA frameworks. For CO_2_ separation, rapid transport of the absorbed CO_2_ in the zeolite is important.^4^ We therefore plotted the parasitic energy *versus* the diameter of the largest sphere that can move through the zeolite using the web tool^9^ provided in ref. 7. From this plot, partly reproduced in Fig. 1, we selected the theoretical frameworks 8124767 and 8277563, as these form the Pareto front: no other theoretical zeolites have both a lower parasitic energy and a larger maximum free sphere.

Zeolites are typically prepared by a hydrothermal synthesis route^10^ in the presence of organic structure directing agents (OSDAs) that promote the formation of the desired zeolite framework.^11,12^ The OSDAs promote the nucleation and growth of the zeolite, and their structure directing capability is correlated with the non-bonded interaction of the OSDA and the zeolite.^13,14^ Thus, the effectiveness of a given molecule to act as an OSDA toward a given zeolite can be predicted using molecular modeling.^15,16^ We have developed a *de novo* design program for the computational design of OSDAs^15^ that has led to the synthesis of a number of zeolites.^17–20^ In the present paper, we describe the application of this *de novo* design program to the design of OSDAs for zeolites that are promising for use in CSS.

## Method

Our *de novo* design algorithm used two separate, independent programs: a scoring function and a molecule generator and optimizer. The scoring function calculated the desired properties for the molecules generated by the molecule generator. First, several properties were calculated from the molecular formula. These included the number of rotatable bonds, the presence of atoms other than C, H, or N, the presence of triply bonded C atoms, the ratio of C atoms to charged N atoms, and the ratio of uncharged to charged N atoms. These properties were used as filters with thresholds set as given in Table 1 to values that are typical for known OSDAs.^21^ For molecules that had properties within these thresholds, the 3D coordinates of a low-energy conformation were generated with molecular mechanics, and the molecular volume was calculated. If the molecular volume was below the threshold, the non-bond interaction between the putative OSDA and the given zeolite framework was calculated using the computational protocol detailed in ref. 15. First, a number of copies of the OSDA was fitted into the zeolite. Subsequently, three molecular dynamics runs were carried out at different time steps, and the average energy of the last five ps of the last dynamics run was used to calculate the OSDA stabilization energy. The latter was defined as *E* = *E*_zeolite:OSDA_ − *E*_zeolite_ − *n* × *E*_OSDA_. *E*_zeolite:OSDA_ is the energy of the zeolite:OSDA complex, *E*_zeolite_ is the energy of the uncomplexed zeolite, *E*_OSDA_ is the energy of the free OSDA, and *n* is the number of copies of the OSDA that were fit into the zeolite. The number of OSDA copies that fit into the zeolite depended on the particular framework and was determined by geometry and trial and error. The zeolite structures were all-Si, and the frameworks were downloaded from the IZA database^6^ or from the database of theoretical zeolites in ref. 8.

**Table 1:** Score vector of molecular properties

Property	Threshold or range
Rotatable bonds	≤5
Non-C, H or N atoms	0
triply bonded C	0
C to N^+^ ratio	4–18
N to N^+^ ratio	≤2
Volume	≤800 Å^3^
Stabilization energy	To be minimized

The *de novo* molecule generator and optimizer was a genetic algorithm (GA) that generates combinations of well documented organic chemistry reactions and reagents from a database of commercially building blocks that give rise to molecules that score well in the above described scoring function.^22^ Currently, a list of 100 reaction has been implemented in the program. However, because no atoms other than C, H, or N were allowed, a number of reactions were turned off, *e.g.* reactions that produce ethers or carbonyl compounds. The total number of available reactions was 42; they are listed in Table S1.† The reagents that could participate in the reactions were organized as a ‘shelf’. We have used a number of such shelves. The shelf with the largest number of compounds was obtained from the Market Select database of the Aldrich company.^23^ To limit the complexity and size of the reagents, only compounds with 15 or fewer heavy atoms were selected. In addition, shelves with molecules containing up to 10, 9, 8, 7, 6, and 5 heavy atoms were created. Another database of reagents was obtained from the building block database of the Aldrich company, with the restriction that these molecules should not have more than one flexible bond.^24^ At the start of the *de novo* design program, a population of virtual synthesis routes was generated randomly, and the score vector of the resulting molecule was calculated by the score function. The number of steps in each synthesis route was also random, but was limited in order to reduce the complexity of the synthesis. For the initial generation of the population this limit was three, and during the GA evolution of the population it was increased to five. Once the population was complete, it was Pareto sorted. The Pareto optimal fronts were calculated, and the lowest fronts were ranked first. Each Pareto front consisted of molecules that did not score better on all scores in the score vector than any molecule in the same front. Next, new molecules and synthesis routes were generated by applying one of six genetic operators to the members of the population. These operators were

• Add a reaction step to a synthesis route.

• Delete a reaction step to a synthesis route.

• Replace a reagent in a synthesis route by a randomly chosen other reagent.

• Replace a reagent in a synthesis route by reagent that is similar.

• Combine two synthesis routes.

• Generate a completely new synthesis route.

The first four operators required the selection of one ‘parent’ synthesis route, and the combine operators needed two parent synthesis routes. These were selected from the Pareto sorted population of synthesis routes by tournament selection: two synthesis routes were picked at random, and the one ranked highest in the population was selected. The resulting new molecule was evaluated by the scoring function, and if it scored better than the worst scoring molecule in the population the latter was replaced by the new molecule. Otherwise it was discarded. After insertion of a new molecule, the populated was re-sorted in a Pareto way. Within each Pareto front, the molecules were sorted according to the order in which they were generated by the GA such that within the same Pareto front, newer molecules had a higher probability to be picked in the tournament selection than older ones. The population was evolved until a total of 200 000 synthesis routes had been evaluated by the scoring function. Details of the *de novo* design algorithm can be found in ref. 24.

Principal coordinate plots^25^ were constructed from the FP2 fingerprint Tanimoto coefficients of the molecules generated by the babel program.^26^

The molecular dynamics was carried out in the NVT ensemble in three stages. All stages were performed at a temperature of 343 K. Before the molecular dynamics, four minimizations were done using alternating stages of Broyden–Fletcher–Goldfarb–Shanno and conjugate-gradient minimization. The first molecular dynamics stage ran for 0.01 ps with a temperature friction coefficient of 0.01 and was used to further optimize the structure. The second molecular dynamics stage ran for 0.1 ps with a temperature friction coefficient of 0.1, again further refining the structure. The third and final molecular dynamics stage ran for 30 ps with a temperature friction coefficient of 0.1. Data for the stabilization energy were collected from the last 5 ps of this third molecular dynamics stage.

## Results

For the five IZA and two theoretical zeolite frameworks, multiple runs were carried out, with varying numbers of OSDA copies and different shelves. The results are shown in Table S2.† The unit cell of the theoretical zeolite 8124767 has a small value for the beta angle, 27° and a short *c*-axis, 9.2 Å. Therefore, in a number of runs the unit cell was expanded along the *c*-axis. Also for the IZA structure JBW, better scoring OSDAs were obtained with an expanded unit cell. A total of 52 runs were carried out on the different frameworks. Table S2† lists the framework identifiers, the number of OSDA copies, the shelf used, the stabilization energy of the best scoring OSDA found, the number of MD runs carried out during the run, the number of OSDAs found with a stabilization energy below 0 kJ (mol Si)^−1^, the number of OSDAs found with a stabilization energy within 2 kJ (mol Si)^−1^ from the stabilization energy of the best scoring OSDA, and the assigned name and the structure of the best scoring OSDA.

For the WEI runs and three 8277563 runs, principal coordinates maps for the 100 best scoring molecules of the runs were constructed. These are shown in Fig. 4 and 5. The fractions of the variance contained in the first two principal coordinates are 0.24 and 0.17 for the WEI plot, and 0.29 and 0.17 for the 8277563 plots.

## Discussion

The target zeolites we have designed OSDAs for have been selected based upon their predicted parasitic energies as defined in ref. 7. The parasitic energy takes into account energetic requirements to capture CO_2_ from typical flue gases. The IZA zeolites we have chosen as targets are the five with the lowest predicted parasitic energies: WEI, JBW, GIS, SIV, and DAC. For the theoretical material our choice was additionally determined by the size of the free channels in the zeolites, which is important for efficient diffusion of CO_2_. Our choice of theoretical target frameworks for our OSDA design effort were the two frameworks that are Pareto optimal in the plot of parasitic energy *versus* maximal free sphere shown in Fig. 1, 8124767 and 8277563. The predicted parasitic energies, maximal free sphere diameters, densities, and energies of the seven zeolite frameworks studied are summarized in Table S3.†

WEI is the framework of the naturally occurring mineral weinebeneite, a beryllophosphate.^27^ We found no reports describing synthetic materials with this framework. Bulk zeolite JBW has been synthesized with an Al/Si ratio of 1 : 1 (ref. 28 and 29) and as low-Si material from kaolinite.^30^ Natural zeolites with the GIS framework are gismondine, amicite, garronite, and gobbinsite,^31^ while synthetic Si–Al GIS zeolites are referred to as P zeolites.^5^ We found no reports of syntheses of all-Si GIS zeolite. The SIV framework was first observed in a cobalt aluminophosphate zeolite obtained by a ionothermal synthesis in an ionic liquid.^32^ While recently all-Si zeolites have been produced with this ionothermal route,^33^ we found no reports on the synthesis of an all-silica SIV framework. DAC is the framework of the naturally occurring mineral dachiardite with framework composition Al_5_Si_19_O_48_. Its synthetic counterpart, with the same Al/Si ratio, has been synthesized under hydrothermal conditions of high pressure and with no OSDAs.^34^ Thus, none of the five most promising known zeolites for CSS have been made in all-Si form. None of the predicted structures we consider for CSS have been synthesized. The OSDAs we designed here may be helpful for synthesizing these zeolites in the all-Si form, which computation has predicted to be optimal for CSS.^26^

A majority of known zeolites have been shown to lie along a straight line in the graph of the calculated framework energy above quartz *versus* the framework density.^35^ This plot is reproduced in Fig. S1 in the ESI,† with the five frameworks selected for CSS shown in green. It can be seen that with the exception of WEI, which is the green data point in the upper part of the graph, the frameworks indeed lie close to this line. As the theoretical structures screened by ref. 7 were selected by their position on the energy–density plot, the two theoretical structures we selected for OSDA design also lie close to the straight line.

The shelves of molecules that were used in the various runs are summarized in Table 2. The size of the chemical space in which the *de novo* design program searches can be estimated as follows. If a shelf contains *N* molecules, and in one synthesis step *n*_0_ zeroth-order and *n*_1_ first-order compounds can be formed, the number of compounds that can be formed in *M* reaction steps can be estimated as *N* × ((*n*_0_ + *n*_1_)/*N*)^*M*^. No reactions requiring more than two reagents have been implemented in the *de novo* design program. The estimated sizes of the search spaces defined by the different shelves with five synthesis steps are summarized in Table 2. It can be seen that even for the smallest shelf used, the Market Select shelf with five or fewer heavy atoms, the search space is far too large to be amenable to an exhaustive search. Nevertheless, it can be seen in Table S2† that even for the largest search space, favorable scoring putative OSDAs were found for each target zeolite framework.

**Table 2:** Number of molecules and estimated size of the chemical search space in the shelves. The shelf names are indicated as MSn for the Market Select shelves with up to *n* heavy atoms, and 1tor for the Aldrich shelf of molecules with zero or one rotatable bonds

Shelf	# of molecules	0^th^ order compounds (one reagent)	1^st^ order compounds (two reagents)	Search space
MS05	597	8.020 × 10^2^	8.455 × 10^4^	3.565 × 10^13^
MS06	2076	2.822 × 10^3^	6.027 × 10^5^	4.384 × 10^15^
MS07	5995	1.001 × 10^4^	3.558 × 10^6^	4.479 × 10^17^
MS08	14 974	2.598 × 10^4^	2.244 × 10^7^	1.138 × 10^20^
MS09	34 021	6.105 × 10^4^	1.211 × 10^8^	1.949 × 10^22^
MS10	69 524	1.342 × 10^5^	5.294 × 10^8^	1.782 × 10^24^
MS15	719 031	1.365 × 10^6^	5.902 × 10^10^	2.681 × 10^30^
1tor	10 180	1.778 × 10^4^	2.337 × 10^7^	6.514 × 10^20^

The results of the different design runs are summarized in Table S2.† As the *de novo* design program is based upon a stochastic algorithm, we strove to perform three duplicate design runs on each target framework. For some frameworks we chose to perform more runs to explore the number of OSDA copies needed, the size of the zeolite unit cell, and the use of an appropriate database of reagents. We first discuss the results on the two theoretical frameworks and then the five IZA frameworks. The IZA frameworks are listed in order of decreasing parasitic energy of the frameworks.

We first designed OSDAs for the theoretical framework 8124767. We performed three runs with the 1tor shelf, trying to fit three and four OSDA per unit cell (rows 1 through 3 in Table S2†). These runs generated molecules with stabilization energies around −3 kJ (mol Si)^−1^, which is unsatisfactory. As the monoclinic unit of this framework has a small beta angle (26°) and a short *c* axis (9.2 Å) relative to the a and *b* axes (15.5 Å and 27.8 Å, respectively), we expanded the unit cell along the *c* axis. Using the same 1tor shelf and fitting between two and six OSDA copies, we still could generate no favorably scoring molecules (rows 4 through 7 of Table S2†). We then switched to the MS05 shelf, which contains molecules with five or fewer heavy atoms collected from the Market Select data set.^23^ With this reagent database we found molecules with stabilization energies lower than −7 kJ (mol Si)^−1^, with four OSDA copies in the non-expanded unit cell framework (rows 8 through 10 in Table S2†). It can be seen in the table that the best scoring OSDAs generated in the second and third of these runs are identical, and that the best scoring OSDA in the first run is very similar to these.

Switching to the MS10 shelf (row 11 in Table S2†) did not produce any molecules with a stabilization energy below 0 kJ (mol Si)^−1^. Using the MS10 shelf in combination with an expanded unit cell also did not generate favorably scoring OSDAs (row 12 in Table S2†). There is a correlation between the success of the *de novo* design program performance and the size of the reagent database, and therefore the search space of the algorithm: with the smaller database containing 597 reagents, relatively favorably scoring molecules can be found, while with the larger databases containing 10 180 (1tor) and 719 031 (MS10) molecules, very few or no molecules scoring below 0 kJ (mol Si)^−1^ can be generated for this zeolite. Even with the smaller shelf, very few acceptable molecules were found, indicating that the 8124767 framework does not easily host guest molecules in comparison to the other frameworks we targeted (*vide infra*).

For the 8277563 framework, we started runs with the MS05 shelf and eight OSDA copies (rows 13 through 19 in Table S2†). This gave rise to designed OSDAs with stabilization energies just above −10 kJ (mol Si)^−1^, and in each run roughly 100 molecules scored below 0 kJ (mol Si)^−1^. To study the effect of the shelf and reagent size we performed runs with sub-shelves of Market Select containing molecules of up to 6, 7, 8, 9 and 10 heavy atoms. In contrast to the 8124767 framework, for the 8277563 framework increasing the search space of the *de novo* search increases the number of molecules scoring below 0 kJ (mol Si)^−1^ from roughly 100 with the MS05 shelf to 366 with the MS09 and MS10 shelves. This behavior is more typical. The score of the best scoring molecule however shows only a modest improvement, from −9.9 kJ (mol Si)^−1^ to −11.1 kJ (mol Si)^−1^. Fig. S2† shows the number of molecules generated below 0 kJ (mol Si)^−1^*versus* the number of molecules in the different shelves.

The best scoring molecules found with the MS05 shelf are identical or very similar for runs 15 and 18, and for runs 14, 16 and 10. Moreover, the best scoring molecules in the latter runs are identical to the two best scoring molecules on the 8124767 framework. This would make them inappropriate as OSDAs specifically targeted towards either framework, see however the discussion below. The MS05 shelf is the smallest database of reagents we used, but even with the much larger shelves MS08 and MS09 two runs converged toward an identical optimal structure, as can be seen in columns 22 and 23 of Table S2.†

The 8277563 framework is characterized by a very large central channel along the *c* axis, formed from 16 SiO_4_ tetrahedra, and a much smaller channel along the 110 direction. The OSDAs with eight copies fitted into the framework only occupy the large channel and may therefore be less suited as templating agents, behaving more as pore-filling agents. We therefore decreased the number of fitted OSDAs to four and two copies. Three runs with four OSDA copies using the MS05 shelf, and three similar runs with the MS10 shelf, generated molecules that scored equally well as the corresponding 8-copy runs. Upon inspection of the generated OSDA–zeolite complexes, we noticed that the larger 4-copy OSDAs have side chains that enter the 110 channel and may therefore be better templates for the 8277563 framework. Decreasing the number of OSDA copies to two produced even larger molecules, but these had a less favorable stabilization energy (row 31 in Table S2†).

**Fig. 2:**
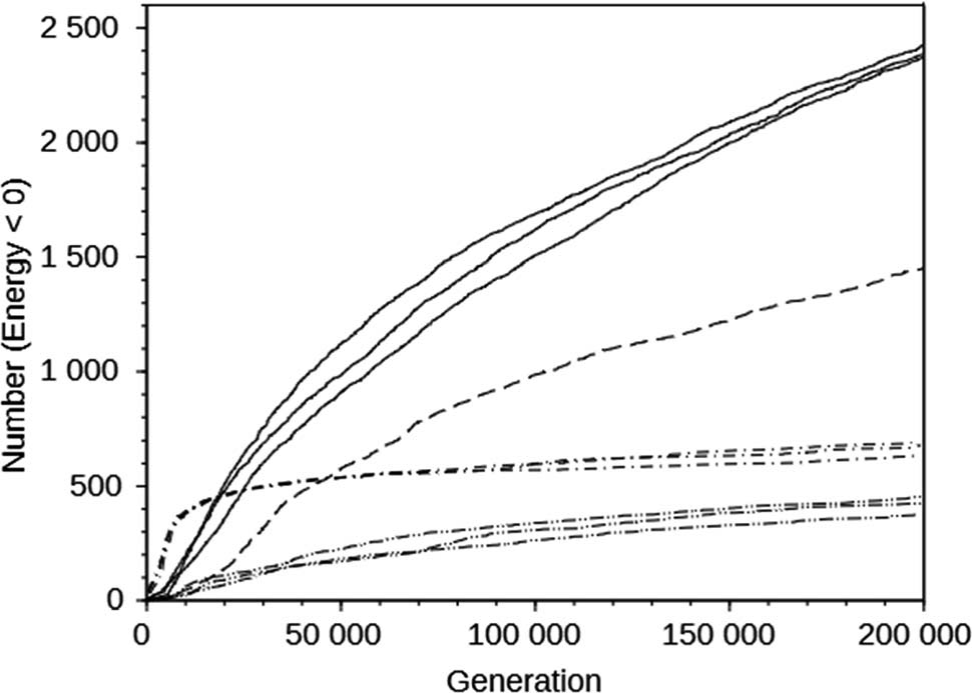
Number of designed molecules with a stabilization energy below 0 kJ (mol Si)^−1^ as a function of the number of molecules generated in the course of the *de novo* design runs on the DAC framework using the MS10 shelf (solid line), the MS15 shelf (dashed line), the MS05 shelf (dot-dashed line), and on the GIS framework with the MS10 shelf (double-dot-dashed line).

For the DAC framework, we performed three runs with two OSDA copies with the MS05 and MS10 shelves and one run with a larger shelf, M15 (rows 32 through 37 in Table S2†). The MS05 runs gave good results, but the MS10 runs produced better best scoring OSDAs and a greater number of molecules scoring below 0 kJ (mol Si)^−1^. However, increasing the number of molecules and the size of the molecules in the shelf in the MS15 run reduced the effectiveness of the algorithm, as judged by the score of the best designed OSDA and the number of molecules with a stabilization energy below 0 kJ (mol Si)^−1^. This is illustrated in the seven upper curves in Fig. 2, showing the cumulative number of molecules with a stabilization energy below 0 kJ (mol Si)^−1^ as a function of the total number of molecules generated. The MS05 runs on DAC initially very quickly generated favorably scoring molecules, but saturated generating novel molecules scoring below 0 kJ (mol Si)^−1^ after ∼15 000 generations. The MS10 runs initially generated favorably scoring molecules more slowly, but kept doing so until the run was ended after 200 000 generations. The MS15 run generated favorably scoring molecules most slowly in the beginning, but continued to do so, although at a slower pace than the MS10 runs. The number of reagents in the MS05, MS10 and MS15 shelves are 597, 69 524, and 719 031, respectively. The above observations are the result of both the size of the search space defined by the size of the shelves and the maximal size of the reagents in the shelves. The MS15 shelf has the MS10 shelf as a subset, and therefore in principle the design should find equally high scoring OSDAs as with the MS10 shelf. Apparently, the much larger search space decreases the efficiency of the GA optimization algorithm. In the search space provided by the MS05 shelf, the number of favorably scoring molecules is smaller, resulting in a less optimally designed OSDA.

For the SIV framework, favorably scoring OSDAs were found when eight copies were fitted into the zeolite. SIV is characterized by a main channel along the 100 direction, and a channel zig–zagging along the 011 and 01̄1 directions. The most favorably scoring OSDAs generated in the first two SIV runs (rows 39 and 40 in Table S2†) mainly occupy the 100 channel, while the significantly better scoring OSDA generated in the third run (row 41 in Table S2†) also fills up the 001/011 channel. The latter OSDA may therefore be expected to be more effective for targeting the SIV framework.

For the GIS framework, favorably scoring molecules were found with two OSDA copies fitted in the framework. The second run (row 43 in Table S2†) was less successful with less optimally designed OSDAs and fewer OSDAs with negative stabilization energies.

A first run on the JBW framework using a single OSDA copy was unsuccessful (row 45 in Table S2†). As this structure has a relatively small unit cell with *a*, *b*, and *c* axes of 5.3 Å, 7.5 Å, and 8.2 Å, respectively, we expanded the unit cell along the shortest (*a*) axis. With the expanded unit cell we designed OSDAs with stabilization energies of −8.4, −8.3, and −13.7 kJ (mol Si)^−1^ (rows 46 through 48 in Table S2†). The best scoring molecules in the first two runs are identical. The third run generated a significantly more optimally designed OSDA. Increasing the number of OSDA copies did not generate favorably scoring molecules (row 49 in Table S2†). In comparison to runs on other frameworks, the JBW framework runs produced very few molecules with negative stabilization energies (column 7 of Table S2†). This may indicate that JBW, as 8124767, does not easily accommodate guest molecules.

**Fig. 3:**
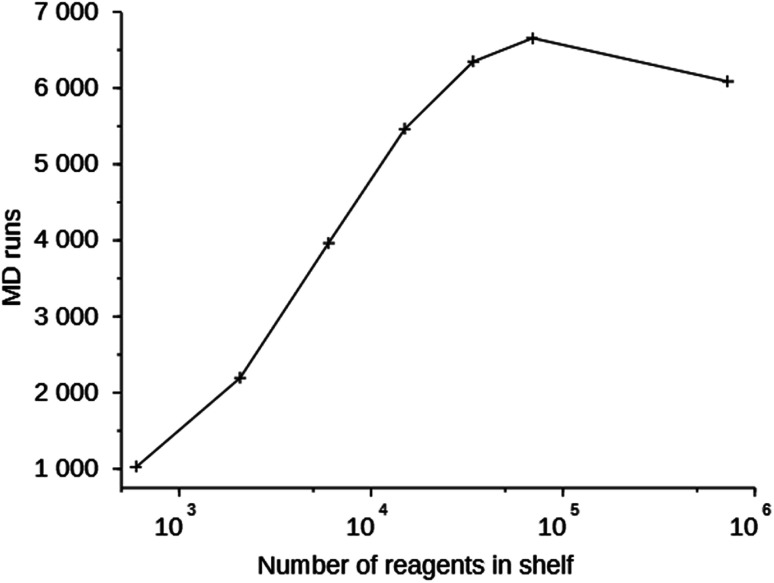
Number of MD evaluations *versus* number of reagents in the shelves in the 8277563 runs using shelves MS05 through MS15.

Column 6 of Table S2† shows the number of MD simulations that has been performed in each run. This number indicates the number of molecules that were generated in 200 000 score function evaluations that passed the simple 1D and 2D filters. It can be seen that this number increases with the size of the space search, an exception being the one run with the MS15 shelf. The MS10 shelf is optimal for most zeolites. It is large enough to be able to produce a variety of OSDAs, yet not so large that the GA cannot effectively search it. Fig. 3 shows the number of MD evaluations *versus* the number of reagents in the shelves used in seven runs on the 8277563 framework. In a larger chemical space it initially is easier for the algorithm to find molecules that fulfill the 1D and 2D constraints, but as the search space becomes very large, the curve inverts.

**Fig. 4:**
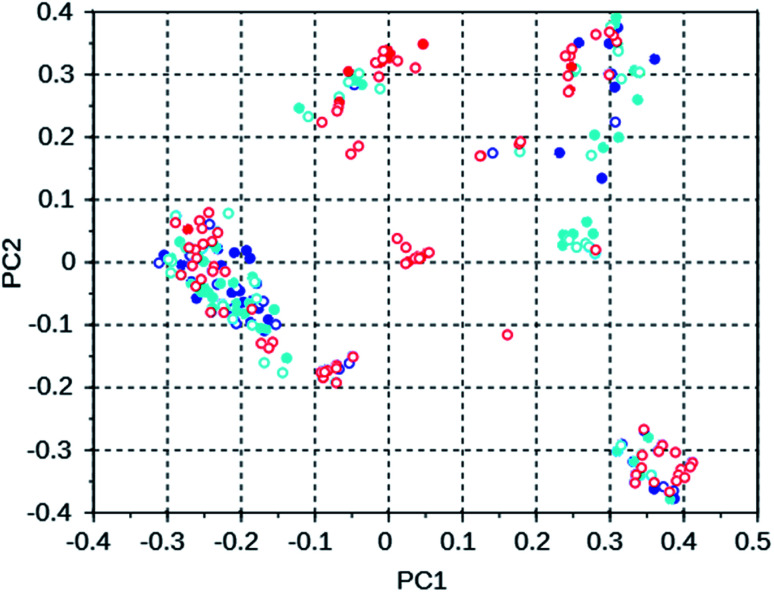
Principal coordinates of the 100 most favorable OSDAs designed in three WEI runs. Blue = run 1, cyan = run 2, red = run 3. The filled circles are from OSDAs with a stabilization energy within 3 kJ (mol Si)^−1^ of the most favorable OSDA, the open circles are from molecules with a less favorable stabilization energy.

To investigate the manner in which the *de novo* design program explores the search space defined by the available set of reactions and the database of reagents, we discuss three runs on the WEI framework, and three runs on the 8277563 framework. The results of these runs can be found in rows 50 through 52 and 28 through 30 of Table S2.† All of these runs used the MS10 shelf of reagents. The first two WEI runs designed OSDAs with stabilization energies of −16.4 and −16.3 kJ (mol Si)^−1^, respectively. The third run designed an OSDA with a stabilization energy of −18.6 kJ (mol Si)^−1^, and the next 8 ranked molecules also have significantly more favorable stabilization energies than any OSDAs in the first two runs. It seems therefore that the GA of the first two *de novo* design runs was stuck in a less optimal part of chemical space. Table 3 lists the number of molecules with a stabilization energy below −10 kJ (mol Si)^−1^ and the overlap between the sets of molecules generated in these runs. Fig. 4 shows a principal component plot based upon the 2-D similarities between the 100 highest ranked molecules in the three runs, with the designed OSDAs having a stabilization score within 3 kJ (mol Si)^−1^ from the best scoring molecule in their run shown as filled dots, and the less optimal OSDAs shown as open circles. Table 3 shows that there is considerable overlap between the designed OSDAs in the three runs. Also, Fig. 4 suggests that these OSDAs can be in clusters in different areas of the search space. The OSDAs designed in the better run however cannot be pinpointed in one particular cluster that is apart from the clusters explored by the other runs.

**Table 3:** Overlap between the 3 runs on the WEI framework. The diagonal entries lists the number of designed OSDAs with a stabilization energy below −10 kJ (mol Si)^−1^, and the off-diagonal entries show the number of OSDAs in common between the runs

	WEI run 1	WEI run 2	WEI run 3
WEI run 1	539	166	173
WEI run 2		543	125
WEI run 3			591

**Fig. 5:**
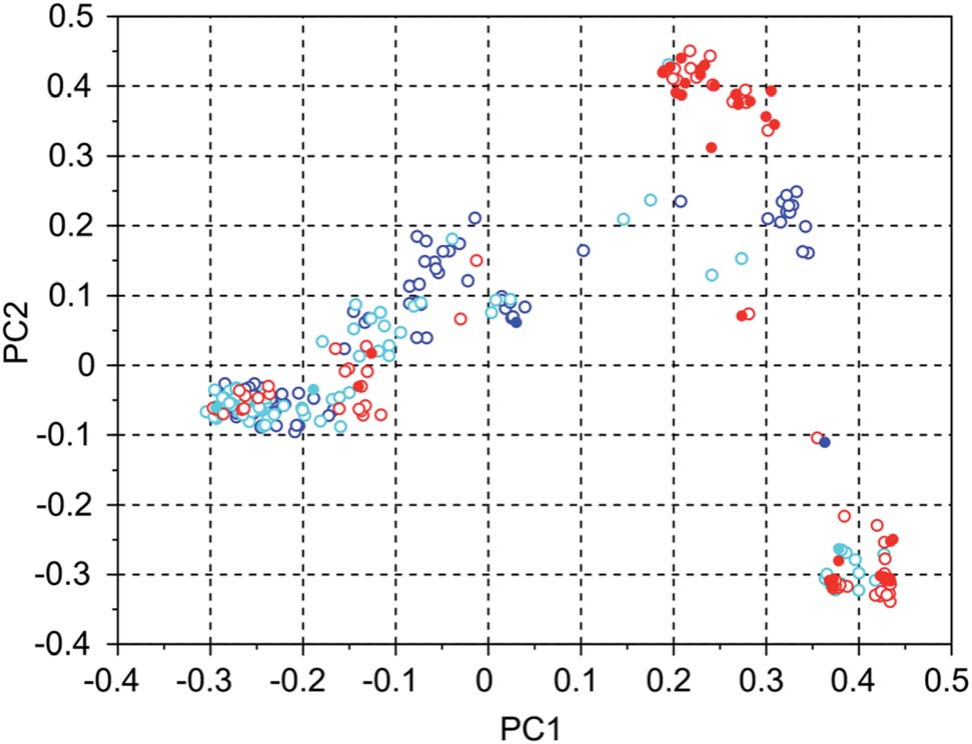
Principal coordinates of the 100 most favorable scoring OSDAs generated in three 8277563 runs with the MS10 shelf and four OSDA copies. Blue = run 1, cyan = run 2, red = run 3. The filled circles are from OSDAs with a stabilization energy lower than −11 kJ (mol Si)^−1^, the open circles are from molecules with a less optimal stabilization energy.

Table 4 shows the number of molecules in the three selected 8277563 runs with a stabilization energy below −10 kJ (mol Si)^−1^ and their overlap. In comparison to the WEI runs, the number of molecules with a stabilization energy below −10 kJ (mol Si)^−1^ is significantly lower, and the overlap between the runs is equally, and also proportionally, smaller. The best scoring molecules in these runs have approximately equal stabilization energies of −11.1, −11.1 and −11.8 kJ (mol Si)^−1^, but the third run has found significantly more molecules having a score below a threshold of −11 kJ (mol Si)^−1^ in the set of 100 best scoring molecules: 34 *versus* 2 and 3. This is also apparent in Fig. 5 of the principal coordinates of the 3 runs, where the molecules with a stabilization energy below −11 kJ (mol Si)^−1^ are highlighted as filled circles. Here, the highest scoring molecules of the third run are mostly concentrated in a cluster that is left almost unexplored in the other two runs.

**Table 4:** Overlap between 3 runs on the 8277563 framework. The diagonal entries lists the number of designed OSDAs with a stabilization energy below −10 kJ (mol Si)^−1^, and the off-diagonal entries show the number of OSDAs in common between the runs

	8277563 run 1	8277563 run 2	8277563 run 3
8277563 run 1	117	13	1
8277563 run 2		113	3
8277563 run 3			288

The upper and lower sets of curves in Fig. 2 show the cumulative number of designed molecules with a stabilization energy below 0 kJ (mol Si)^−1^ for the most productive DAC and GIS runs using the MS10 shelf. The number of favorably scoring molecules generated in the course of a run differs greatly between the frameworks, but the overall shape of the curves is very similar.

**Fig. 6:**
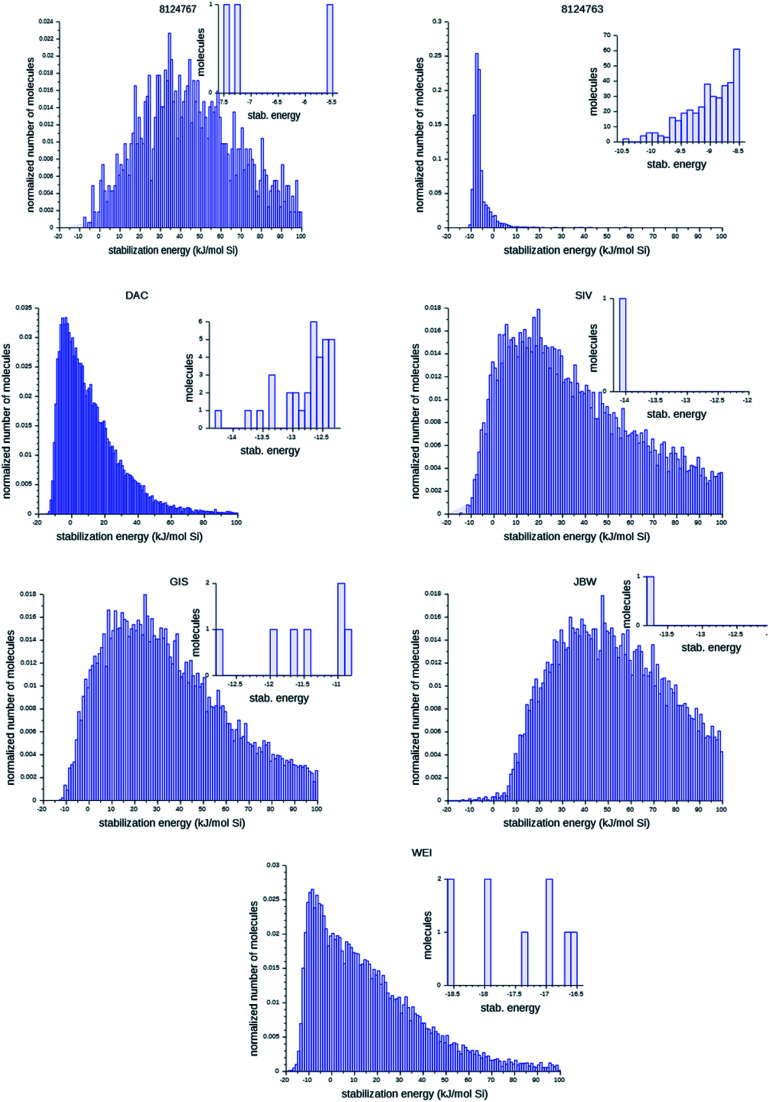
Histograms of the stabilization energies of designed molecules. The large figures present the cumulative results of the three best runs for each target zeolites. The insets show the stabilization energies of the most favorably scoring OSDAs within a range of 2 kJ (mol Si)^−1^ in each run. For 8124763, results for *n* = 4 OSDAs are shown.

Fig. 6 shows the probability distributions of the stabilization energies of the molecules generated by the most successful runs on each target zeolite. In inset are shown the most favorably scoring OSDAs designed; these are the molecules that may control the targeted syntheses of the seven zeolites considered here. There are large variations in the overall shape of the histograms and the number of OSDAs with lowest stabilization energies over the seven target zeolites: for the 8277563 framework, a large number of favorably scoring structures was found. In contrast, for JBW only one favorably scoring OSDA was generated in the three runs, and also for 8124767, SIV, GIS, and WEI the number of optimally designed OSDAs was limited.

From the above analysis, we observe that the way in which the *de novo* design algorithm explored its search space was highly dependent on the number and size of the reagents in the reagent database, and on the characteristics of the target framework. The latter observation is not surprising as the structure of the framework determines the shape of the energy function in this molecular search space.

## Conclusion

We have designed OSDAs that can be helpful in the synthesis of five known and two theoretical zeolites that have been predicted to be effective materials for carbon capture and storage. As the criteria for CSS usefulness, we used the parasitic energy of separating the CO_2_ from power plant exhaust gasses. We designed several OSDAs for each zeolite. We noticed that the effectiveness of the *de novo* design program was highly dependent on both the structure of the targeted zeolite and the size of the chemical search space the algorithm explores. The OSDAs we have designed may aid the synthesis of zeolites that may mitigate global warming.

## Conflicts of interest

Frits Daeyaert is manager of FD Computing. Michael W. Deem is a consultant for the petrochemical industry in the area of zeolites. Those relationships did not affect the design or outcome of the present research.

## Supplementary Material

RA-009-C9RA09675B-s001

RA-009-C9RA09675B-s002

RA-009-C9RA09675B-s003

RA-009-C9RA09675B-s004

RA-009-C9RA09675B-s005

RA-009-C9RA09675B-s006

RA-009-C9RA09675B-s007

RA-009-C9RA09675B-s008
