# Supplementary material for: Design of organic structure directing agents to control the synthesis of zeolites for carbon capture and storage
Source: RSC Adv. 2019 Dec 17;9(71):41934–42. doi: 10.1039/c9ra09675b (PMC9076543; doi:10.1039/c9ra09675b)
Supplement: RA-009-C9RA09675B-s001 [file RA-009-C9RA09675B-s001.pdf]

Electronic Supplementary Material (ESI) for RSC Advances.
This journal is © The Royal Society of Chemistry 2019

Supplementary Information

Table S1: Selection of organic synthesis reactions used by the de novo *design program to generate OSDAs.*

Mnemonic	Reactants	Product	Description
AMIDEFROMACID	R_1C=OOH, R_2NH_2	R_1-NHC=O-R_2	Amide from acid and aliphatic amine
REDUCECYAN	RC#N	R-CNH_2	Red.Am. Fully reduce CN to amine
WITTIG	R_1CXR_2, R_3C=OR_4	$R_{1,2}C$=$CR_{3,4}$	Wittig alkene synthesis
NH2FROMNITRO	RNO_2	RNH_2	Reduce nitro group
AMINEFROMAMIDE	R_1C=$ONR_{2,3}$	$R_1CH_2NR_{2,3}$	Red.Am. Reduce amide group
AMINEFROMACID	RC=OOH	RNH_2	Schmidt Reaction, reduce acid group
AMINEFROMALDOKETO	R_1C=OR_2	$R_1CHNH_2R_2$	Reductive amination from carbonyl
FCACYLATION	Aryl, R_2C=OX	Ar1C=OR_2	Friedel-Crafts acylation
DEOXYALDOKETO	R_1C=OR_2	$R_1CH_2R_2$	Aldol/Ketone deoxgenation
REDALDOKETO	R_1C=OR_2	R_1CHOHR_2	Reduction of aldol/ketone to alchohol
GABRIEL	RX	RNH_2	Alkyl-NH2 from halide
ALKYNECOUPLING	R_1C#CH, R_2C#CH	R_1C#CC#CR_2	Alkyne coupling
APPEL	ROH	RX	Appel reaction
BARTOLIINDOLE	R_1NO_2Phe, XR_2C=CR_3	$R_{1,2,3}$ Indole	Bartoli indole synthesis
BUCHWALDHARTWIG	ArX, R_1NHR_2	$ArNR_{1,2}$	Buchwald Hartwig coupling
ALKENETOALKANE	$R_{1,2}C$=$CR_{3,4}$	$R_{12}CHCHR_{34}$	Reduction of alkene
COREYFUCHS	RHC=O	RC#CH	Corey-Fuchs alkyne synthesis
ALKYNETOALKENE	R_1C#CR_2	R_1HC=CHR_2	Reduction of alkyne to alkene
ALKYNETOALKANE	R_1C#CR_2	$R_1H_2CCH_2R_2$	Reduction of alkyne to alkane
ALKYNESN2	R_1C#CH, R_2X	R_1C#CR_2	SN2 displacement of alkyne
OHTOBR	ROH	RBr	Bromide synthesis
OHTOI	ROH	RI	Iodide synthesis
BENZYLTOCHLORIDE	$ArCH_3$	$ArCH_2Cl$	Benzyl halogenization
BENZYLTOBROMIDE	$ArCH_3$	$ArCH_2Br$	Benzyl halogenization
HALOTOCYANIDE	RX	RC#N	Cyanide from halide
SONOGASHIRA	R_1C#CH, R_2X	R_1C#CR_2	Sonogashira coupling
LAROCKINDOLE	$ArINHR_1$,R_2C#CR_3	R_{123}indol	Larock indole synthesis
SUZUKI	borate, halogenide	Alkyl/$aryl_1$-Alkyl/$aryl_2$	Suzuki coupling
NHALKYLATION	aromatic N-H, RX	ArN-R, ArN = imidazole, ...	Alkylation of arom. N with Grignard
FISCHERINDOLE	Hydrazine, carbonyl	indole	Fischer indole synthesis
MENSHUTKIN	R1R2R3N, R4X	Quaternary amine	Menshutkin reaction
SKRAUP	anyline with alpha-CH	quinoline	Skraup reaction
SUZUKIMIYAURA	2x Alkyl/aryl -X	Alkyl/$aryl_1$-Alkyl/$aryl_2$	Suzuki-Miyaura reaction
ESCHWEILERCLARKE	prim./sec. amine	methyl-substituted amine	Eschweiler-Clarke reaction
PYRIMIDINIUM	pyridine, SN2-halogenide	pyrimidinium cation	alkylated pyridine (pyridinium ion)
ALKYLATEN	amine, MeI or EtI	methyl or ethyl amine	fully methylate or ethylate an amine

Mnemonic	Reactants	Product	Description
KNORRPYRAZOLE	RC(=O)CC(=O)R, Hydrazin	Pyrazole	Knorr Pyrazole synthesis
ALKYLATENP	aromatic N, SN2 halegonide	N-alklated aromatic heterocycle	Alkylation of aromatic nitrogen
CXBORYLATION	prim./sec. halogenide	alkyl-$B(OH)_2$	Boronation of halogenide
ALKYLATESECAM	sec. amine	prim./sec. halogenide	Alkylation of sec. amine
ALKYLATENPORNPH	aromatic N or NH	prim./sec. halogenide	Alkylation of aromatic amine
PYRIDINETOC	pyridine	phenyl equivalent	Replace pyridine with phenyl

*Table S2: Results of the 52 d*e novo *design runs to generate OSDAs for the seven target zeolites. The nine columns display the number of the run (column 1), the name of the target zeolite (column 2), the number of OSDA copies fitted in the target zeolite (column 3), the shelf used (column 4), the stabilization energy of the most favorable scoring molecule found in the run (column 5), the number of MD simulations carried out in the run (column 6), the number of OSDAs found with a stabilization energy below 0 kJ/(mol Si) (column 7), the number of OSDAs found with a stabilization energy within 2 kJ/(mol Si) from the stabilization energy of the most favorably scoring OSDA (column 8), and the identifier and 2D structure of the most favorably scoring OSDA found in the run (column 0). If an expanded unit cell was used in a run, this is indicated in parenthesis in column 2.*

run	target	osda copies	shelf	best score	MD runs	<0 kj/(mol Si)	within 2 kJ/(mol Si)	best OSDA
1	8124767	3	1tor	-3.0 kJ/(mol Si)	3198	12	10	Syn004317 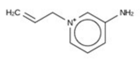
2	8124767	4	1tor	-0.5 kJ/(mol Si)	3429	1	4	Syn040158 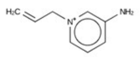
3	8124767	4	1tor	-0.6 kJ/(mol Si)	3926	2	10	Syn018621 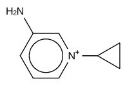
4	8124767 (112)	2	1tor	-3.1 kJ/(mol Si)	4198	376	125	Syn041716 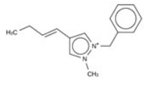

run	target	osda copies	shelf	best score	MD runs	<0 kj/(mol Si)	within 2 kJ/(mol Si)	best OSDA
5	8124767 (112)	3	1tor	-3.5 kJ/(mol Si)	3781	199	54	Syn020911 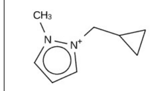
6	8124767 (112)	4	1tor	-3.5 kJ/(mol Si)	3524	68	23	Syn010119 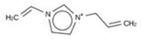
7	8124767 (112)	6	1tor	-3.8 kJ/(mol Si)	3319	15	4	Syn040165 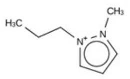
8	8124767	4	MS05	-7.2 kJ/(mol Si)	1089	13	1	Syn034587 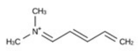
9	8124767	4	MS05	-7.4 kJ/(mol Si)	1189	13	1	Syn014680 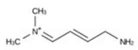
10	8124767	4	MS05	-7.3 kJ/(mol Si)	1248	13	1	Syn043731 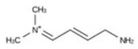
11	8124767	4	MS10	0.4 kJ/(mol Si)	6301	0	9	Syn019677 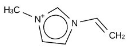
12	8124767 (112)	4	MS10	-4.4 kJ/(mol Si)	7144	232	22	Syn094775 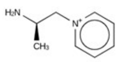
13	8277563	8	MS_05	-9.9 kJ/(mol Si)	1240	113	11	Syn003616 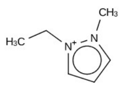

run	target	osda copies	shelf	best score	MD runs	<0 kj/(mol Si)	within 2 kJ/(mol Si)	best OSDA
14	8277563	8	MS_05	-9.4 kJ/(mol Si)	1381	123	12	Syn018987
15	8277563	8	MS_05	-9.8 kJ/(mol Si)	1131	102	13	Syn003646 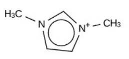
16	8277563	8	MS_05	-9.6 kJ/(mol Si)	1160	101	15	Syn119920
17	8277563	8	MS_05	-9.9 kJ/(mol Si)	1240	113	14	Syn003616 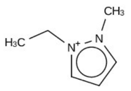
18	8277563	8	MS_05	-9.5 kJ/(mol Si)	1160	114	13	Syn007701 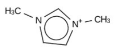
19	8277563	8	MS_05	-9.9 kJ/(mol Si)	1024	95	10	Syn006053 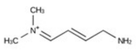
20	8277563	8	MS_06	-10.3 kJ/(mol Si)	2193	168	11	Syn004112 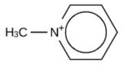
21	8277563	8	MS_07	-11.1 kJ/(mol Si)	3963	318	15	Syn011234 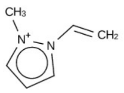
22	8277563	8	MS_08	-10.7 kJ/(mol Si)	5461	285	25	Syn001729

run	target	osda copies	shelf	best score	MD runs	<0 kj/(mol Si)	within 2 kJ/(mol Si)	best OSDA
23	8277563	8	MS_09	-10.5 kJ/(mol Si)	6346	366	28	Syn061594
24	8277563	8	MS_10	-11.1 kJ/(mol Si)	6655	366	11	Syn144424
25	8277563	4	MS_05	-9.9 kJ/(mol Si)	1271	1189	238	Syn022105 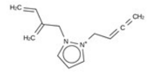
26	8277563	4	MS_05	-10.4 kJ/(mol Si)	1669	1556	238	Syn039747 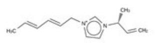
27	8277563	4	MS_05	-10.5 kJ/(mol Si)	1805	1686	225	Syn135934 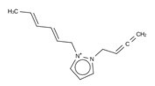
28	8277563	4	MS_10	-11.1 kJ/(mol Si)	6487	5071	755	Syn026580 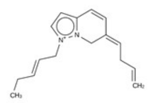
29	8277563	4	MS_10	-11.1 kJ/(mol Si)	7317	5791	876	Syn125982 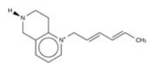
30	8277563	4	MS_10	-11.8 kJ/(mol Si)	7320	5800	448	Syn171369 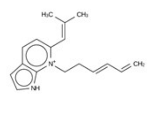
31	8277563	2	MS_10	-9.3 kJ/(mol Si)	6881	6569	3491	Syn175368 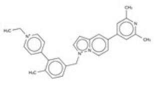

run	target	osda copies	shelf	best score	MD runs	<0 kj/(mol Si)	within 2 kJ/(mol Si)	best OSDA
32	DAC	2	MS_05	-11.3 kJ/(mol Si)	1259	679	22	Syn163734 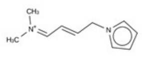
33	DAC	2	MS_05	-11.1 kJ/(mol Si)	1163	633	19	Syn000651 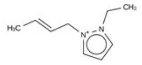
34	DAC	2	MS_05	-11.2 kJ/(mol Si)	1232	691	17	Syn004268 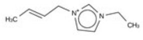
35	DAC	2	MS_10	-13.1 kj/(mol Si)	6787	2424	41	Syn074271 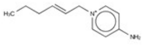
36	DAC	2	MS_10	-14.2 kJ/(mol Si)	6952	2384	20	Syn124337 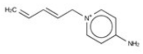
37	DAC	2	MS_10	-13.7 kJ/(mol Si)	7019	2371	25	Syn120443 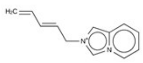
38	DAC	2	MS_15	-12.9 kJ/(mol Si)	6087	1455	17	Syn141805 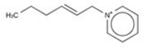
39	SIV	8	MS_10	-11.4 kJ/(mol Si)	6344	516	17	Syn012738 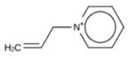
40	SIV	8	MS_10	-11.9 kJ/(mol Si)	4531	456	10	Syn085308 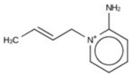
41	SIV	8	MS10	-14.1 kJ/(mol Si)	6533	487	1	Syn170524 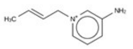

run	target	osda copies	shelf	best score	MD runs	<0 kj/(mol Si)	within 2 kJ/(mol Si)	best OSDA
42	GIS	2	MS_10	-12.0 kJ/(mol Si)	6782	454	14	Syn191726 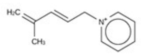
43	GIS	2	MS_10	-10.5 kJ/(mol Si)	7771	376	12	Syn020151 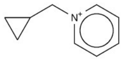
44	GIS	2	MS_10	-12.8 kJ/(mol Si)	5088	425	5	Syn075888 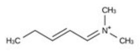
45	JBW	1	MS_10	1.2 kJ/(mol Si)	6647	0	1	Syn014767 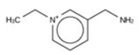
46	JBW (211)	1	MS_10	-8.4 kJ/(mol Si)	7277	6	1	Syn005431 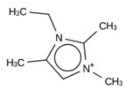
47	JBW (211)	1	MS_10	-8.3 kJ/(mol Si)	7200	7	1	Syn004598 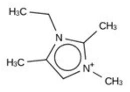
48	JBW (211)	1	MS_10	-13.7 kJ/(mol Si)	6861	8	1	Syn032232 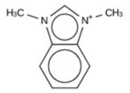
49	JBW (211)	2	MS_10	30.3 kJ/(mol Si)	6686	0	1	Syn121186 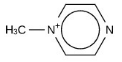

run	target	osda copies	shelf	best score	MD runs	<0 kj/(mol Si)	within 2 kJ/(mol Si)	best OSDA
50	WEI	2	MS_10	-16.4 kJ/(mol Si)	7243	2440	22	Syn129375 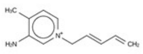
51	WEI	2	MS_10	-16.3 kJ/(mol Si)	8100	2527	18	Syn118769 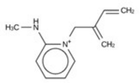
52	WEI	2	MS_10	-18.6 kJ/(mol Si)	6881	2319	9	Syn118466 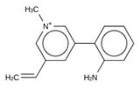

Table S3: IZA and theoretical zeolite frameworks for which OSDAs have been designed in this work.

Framework	Parasitic Energy	Max. free sphere	Density	Energy above quartz
WEI	732.44 kJ/(kg CO_2)	3.40 Å	16.5 T/1000Å^3	34.8 kJ/mol
JBW	746.47 kJ/(kg CO_2)	3.66 Å	18.8 T/1000Å^3	11.0 kJ/mol
GIS	793.66 kJ/(kg CO_2)	3.26 Å	16.4 T/1000Å^3	15.0 kJ/mol
SIV	839.32 kJ/(kg CO_2)	3.67 Å	16.4 T/1000Å^3	15.4 kJ/mol
DAC	841.7 kJ/(kg CO_2)	4.13 Å	17.5 T/1000Å^3	12.3 kJ/mol
8124767	727.96 kJ/(kg CO_2)	3.38 Å	17.8 T/1000Å^3	14.7 kJ/mol
8277563	735.85 kJ/(kg CO_2)	7.53 Å	16.9 T/1000Å^3	14.2 kJ/mol

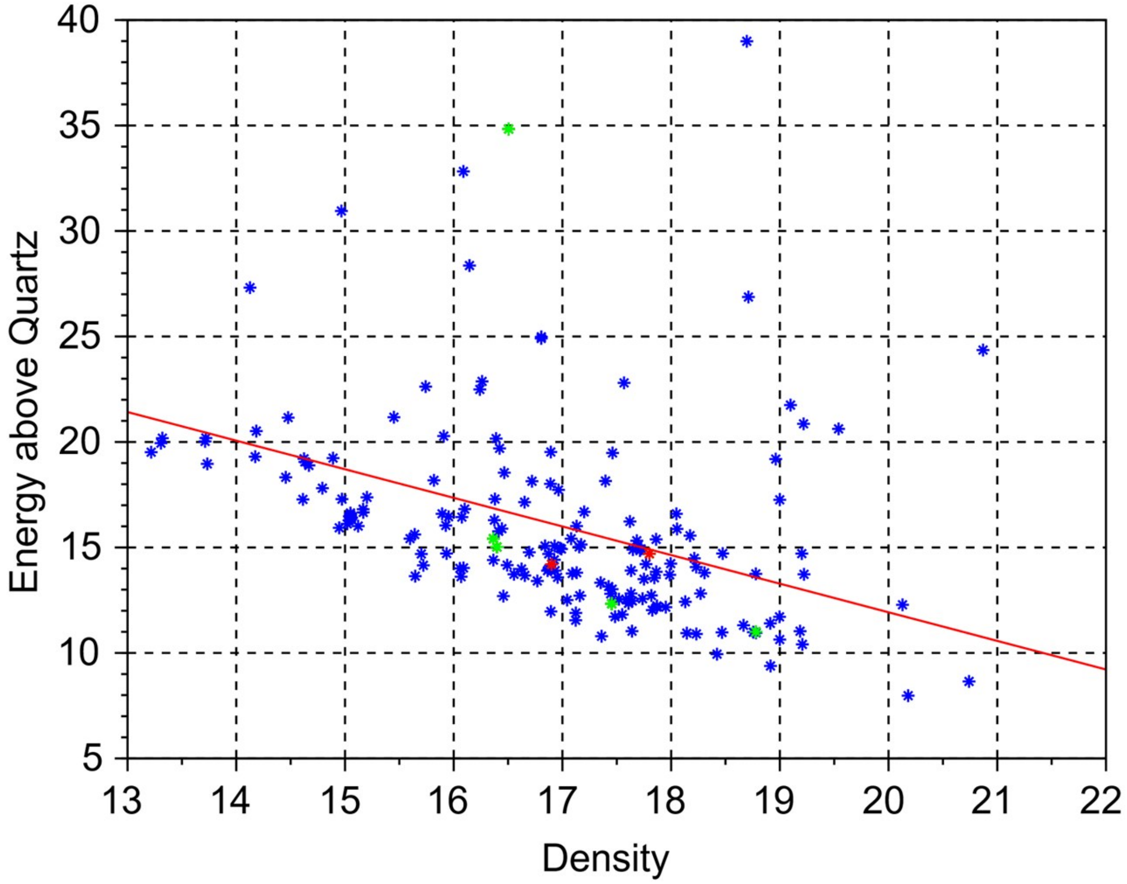

Figure S1: Energy-density plot for the five known target zeolites (green), the two theoretical target zeolites (red), and the other known zeolites with an energy above quartz below 40 kJ/(mol Si) (blue). The energies are in kJ/(mol Si), the densities are in Si atoms per Å^3. The red line is the fit through the energy-density plot of known zeolites.

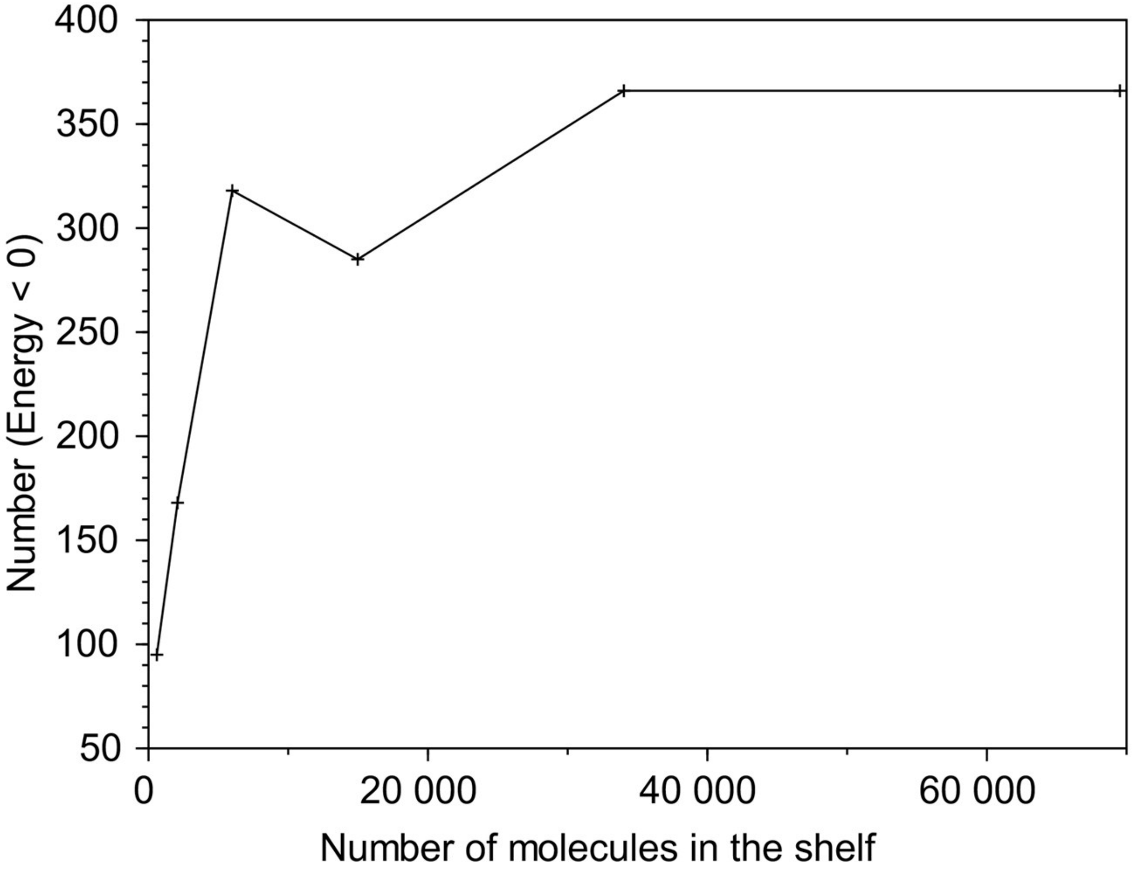

Figure S2: Number of generated OSDAs with a stabilization energy below 0 kJ/(mol Si) versus the number of molecules in the shelf for six runs on 8277563.
